# Supplementary material for: Early neutrophil activation and NETs release in the pristane-induced lupus mice model
Source: PLoS One. 2025 Jan 3;20(1):e0306943. doi: 10.1371/journal.pone.0306943 (PMC11698329; doi:10.1371/journal.pone.0306943)
Supplement: S1 Table — (DOCX) [file pone.0306943.s001.docx]

**Supplementary Table** Number of activated neutrophils, low-density granulocytes, and NETs release by activated neutrophils and low-density granulocytes by each mice group and site.

**

#

Nets -Spleen

*

*

*

**

**

**

Ŧ

&

Nets – Bone Marrow

Nets - Peritoneal lavage

| Site / Group | Basal value (n= 12) | Saline (n=6) | Pristane (n=6) |
| --- | --- | --- | --- |
| Activated neutrophils | | | |
| Peripheral blood | 660.6 ± 478.3  534 (86 – 1,981) | **1,149 ± 183.8**  1,149 (1,019 – 1,279) | **4,120.2 ± 2,648.5**  4,043 (1,411 – 6,862) |
| Peritoneal lavage |  | 569.7 ± 538.4  547 (43 – 1,119) | **4,953 ± 797**  4,947 (3,926 – 6,005) |
| Bone marrow |  | 302.3 ± 48.3  315 (249 - 343) | **6,222.8 ± 515.3**  6,505 (5,332 – 6,590) |
| Spleen |  | 1,739 ± 102.5  1,762 (1,627 – 1,828) | 2,119.7 ± 638.6  2,230.5 (1,173 – 2,778) |
| Low-density granulocytes | | | |
| Peripheral blood | 38.8 ± 37.7  31.5 (0 - 123) | **418 ± 36.8**  444 (392 - 613) | **1,998.3 ± 1,012.4**  2,162.5 (839 - 2924) |
| Peritoneal lavage |  | 448.3 ± 141.5  476 (295 - 574) | **2,813 ± 1,032.8**  2,386.5 (839 - 4197) |
| Bone marrow |  | 168.7 ± 25.9  180 (139 - 187) | **2,483.5 ± 372.2**  2,472.5 (2,079 – 2,922) |
| Spleen |  | 802 ± 65.2  770 (759 - 877) | 929.2 ± 182.7  976.5 (597 - 1140) |
| NETs release by activated neutrophils | | | |
| Peripheral blood | **383.2 ± 423.7**  244 (56 – 1,925) | **393 ± 49.5**  393 (358 - 428) | **3,371.5 ± 2,162.9**  3,350 (1,145 – 5,660) |
| Peritoneal lavage |  | 308.7 ± 285.9  327 (14 - 585) | **2,035.5 ± 636.1**  2,007 (1,352 – 2,767) |
| Bone marrow |  | 154 ± 29.1  152 (126 - 184) | **1,735 ± 639.7**  1,884.5 (789 - 2,523) |
| Spleen |  | 432.7 ± 113.8  397 (341 - 560) | **1,179 ± 299**  1,245.5 (675 - 1471) |
| NETs release by low-density granulocytes | | | |
| Peripheral blood | **5.7 ± 6.8**  2.5 (0 - 26) | **178 ± 29.7**  178 (157 - 199) | **1,623.3 ± 824.3**  1,760 (676 - 2,388) |
| Peritoneal lavage |  | 186.7 ± 42.2  168 (157 - 235) | **1,011 ± 387.5**  942 (637 – 1,448) |
| Bone marrow |  | 93 ± 20.1  91 (74 - 114) | **744.5 ± 275**  778 (390 – 1,100) |
| Spleen |  | 209.7 ± 52.4  183 (176 - 270) | **534 ± 106.4**  538.5 (359 - 666) |

Results are shown by mean, standard deviation, median, minimum, and maximum. *p <* 0.05 compared to the basal value; *p <* 0.05 compared to the saline group.
