# Supplementary figures and images for: Early neutrophil activation and NETs release in the pristane-induced lupus mice model
Source: PLoS One. 2025 Jan 3;20(1):e0306943. doi: 10.1371/journal.pone.0306943 (PMC11698329; doi:10.1371/journal.pone.0306943)

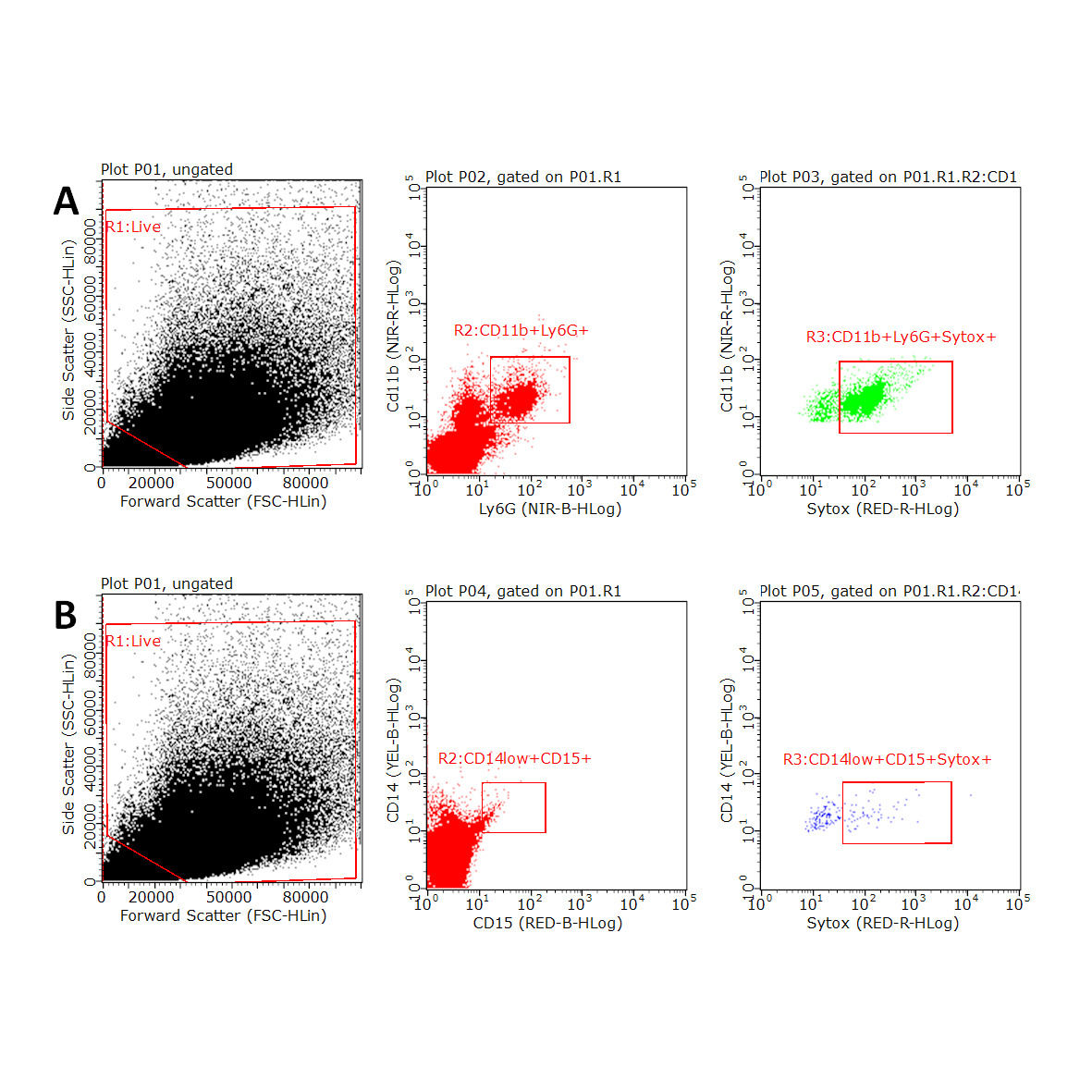

Supplement: S1 Fig — A: Activated neutrophil with sytox positivity. R1 region containing the neutrophil and monocyte populations. Within the R1 region, activated neutrophils were detected by monoclonal antibodies Ly6G and CD11b double-positive R2 region. Within the R2 region, Sytox Green-positive activated neutrophils were quantified by the release of NETs (R3). B: Low density granulocytes with sytox positivity. R1 region containing the neutrophil and monocyte populations. Within the R1 region, low density granulocytes were detected by monoclonal antibodies CD15+CD14low (LDGs) double-positive R2 region. Within the R2 region, Sytox Green-positive LDGs were quantified by NETs release (R3). (TIF) [file pone.0306943.s002.tif]
