# Supplementary material for: Early neutrophil activation and NETs release in the pristane-induced lupus mice model
Source: PLoS One. 2025 Jan 3;20(1):e0306943. doi: 10.1371/journal.pone.0306943 (PMC11698329; doi:10.1371/journal.pone.0306943)
Supplement: S1 Dataset — (DOCX) [file pone.0306943.s003.docx]

| Blood - number activated neutrophil |  |  | number LDGs |  |  | NETs activated neutrophil |  |  | NETs LDGs |  |  |
| --- | --- | --- | --- | --- | --- | --- | --- | --- | --- | --- | --- |
|  |  |  |  |  |  |  |  |  |  |  |  |
| Basal - Ly6G+CD11b | Saline- Ly6G+CD11b | Pristane- Ly6G+CD11b | Basal - CD15+CD14low | Saline- CD15+CD14 low | Pristane- CD15+CD14low | Basal –  Ly6G+CD11b | Saline- Ly6G+CD11b | Pristane- Ly6G+CD11b | Basal number- CD15+CD14low | Saline- CD15+CD14 low | Pristane- CD15+CD14low |
| 280 | 1019 | 5786 | 56 | 392 | 2880 | 251 | 428 | 4826 | 10 | 199 | 2358 |
| 459 | 1279 | 6838 | 89 | 444 | 2909 | 425 | 358 | 5464 | 13 | 157 | 2333 |
| 1199 |  | 6862 | 72 |  | 2924 | 1145 |  | 5660 | 13 |  | 2388 |
| 1981 |  | 1524 | 95 |  | 993 | 1925 |  | 1260 | 26 |  | 798 |
| 918 |  | 1411 | 90 |  | 839 | 873 |  | 1145 | 14 |  | 676 |
| 231 |  | 2300 | 50 |  | 1445 | 196 |  | 1874 | 7 |  | 1187 |
| 406 |  |  | 60 |  |  | 368 |  |  | 7 |  |  |
| 251 |  |  | 66 |  |  | 179 |  |  | 5 |  |  |
| 230 |  |  | 123 |  |  | 163 |  |  | 16 |  |  |
| 155 |  |  | 47 |  |  | 119 |  |  | 5 |  |  |
| 244 |  |  | 73 |  |  | 206 |  |  | 10 |  |  |
| 86 |  |  | 40 |  |  | 56 |  |  | 2 |  |  |
| 870 |  |  | 2 |  |  | 248 |  |  | 0 |  |  |
| 575 |  |  | 7 |  |  | 332 |  |  | 1 |  |  |
| 690 |  |  | 1 |  |  | 240 |  |  | 0 |  |  |
| 935 |  |  | 0 |  |  | 279 |  |  | 0 |  |  |
| 530 |  |  | 4 |  |  | 67 |  |  | 0 |  |  |
| 1350 |  |  | 4 |  |  | 239 |  |  | 1 |  |  |
| 1309 |  |  | 7 |  |  | 839 |  |  | 2 |  |  |
| 803 |  |  | 23 |  |  | 344 |  |  | 3 |  |  |
| 1189 |  |  | 8 |  |  | 248 |  |  | 0 |  |  |
| 538 |  |  | 1 |  |  | 164 |  |  | 0 |  |  |
| 406 |  |  | 2 |  |  | 234 |  |  | 0 |  |  |
| 219 |  |  | 12 |  |  | 56 |  |  | 1 |  |  |
|  |  |  |  |  |  |  |  |  |  |  |  |
|  |  |  |  |  |  |  |  |  |  |  |  |

| Peritoneal lavage - activated neutrophil | Activated neutrophil | | LDGs | LDGs | | Number | |  |  | Bone Marrow - activated neutrophil | | Activated neutrophil | LDGs | LDGs Number |
| --- | --- | --- | --- | --- | --- | --- | --- | --- | --- | --- | --- | --- | --- | --- |
| Saline- Ly6G+CD11b | Pristane- Ly6G+CD11b | | Saline- CD15+CD14 low | Pristane- CD15+CD14low | |  | |  |  | Saline- Ly6G+CD11b | | Pristane- Ly6G+CD11b | Saline-CD15+CD14 low | Pristane- CD15+CD14low |
| 14 | 2462 | | 168 | 1448 | |  | |  |  | 126 | | 2523 | 74 | 1100 |
| 585 | 2590 | | 235 | 1191 | |  | |  |  | 184 | | 2144 | 114 | 785 |
| 327 | 2767 | | 157 | 1425 | |  | |  |  | 152 | | 1795 | 91 | 771 |
|  | 1552 | |  | 637 | |  | |  |  |  | | 1974 |  | 957 |
|  | 1490 | |  | 693 | |  | |  |  |  | | 1185 |  | 464 |
|  | 1352 | |  | 672 | |  | |  |  |  | | 789 |  | 390 |
|  |  | |  |  | |  | |  |  |  | |  |  |  |
|  |  | |  |  | |  | |  |  |  | |  |  |  |
| Peritoneal lavage - activated neutrophil | **Activated neutrophil** | | **LDGs** | **LDGs** | | **NETs** | |  |  | **Bone Marrow - activated neutrophil** | | **Activated neutrophil** | **LDGs** | **LDGs NETs** |
| Saline- Ly6G+CD11b | Pristane- Ly6G+CD11b | | Saline- CD15+CD14 low | Pristane- CD15+CD14low | |  | |  |  | Saline- Ly6G+CD11b | | Pristane- Ly6G+CD11b | Saline- CD15+CD14 low | Pristane- CD15+CD14low |
| 43 | 3926 | | 574 | 4197 | |  | |  |  | 249 | | 6547 | 139 | 2872 |
| 1119 | 4336 | | 476 | 3328 | |  | |  |  | 343 | | 6474 | 187 | 2360 |
| 547 | 4580 | | 295 | 3621 | |  | |  |  | 315 | | 6536 | 180 | 2585 |
|  | 5557 | |  | 993 | |  | |  |  |  | | 6590 |  | 2922 |
|  | 6005 | |  | 839 | |  | |  |  |  | | 5858 |  | 2083 |
|  | 5314 | |  | 1445 | |  | |  |  |  | | 5332 |  | 2079 |
|  |  | |  |  | |  | |  |  |  |  |  |  |  |
| Spleen - activated neutrophil | | **activated neutrophil** | | | **LDGs** | | **LDGs** | | | |  |  |  |  |
| Saline-  Ly6G+CD11b | | Pristane-  Ly6G+CD11b | | | Saline-  CD15+CD14 low | | Pristane-  CD15+CD14low | | | |  |  |  |  |
| 560 | | 1471 | | | 270 | | 613 | | | |  |  |  |  |
| 341 | | 1255 | | | 176 | | 489 | | | |  |  |  |  |
| 397 | | 1437 | | | 183 | | 528 | | | |  |  |  |  |
|  | | 1236 | | |  | | 666 | | | |  |  |  |  |
|  | | 675 | | |  | | 359 | | | |  |  |  |  |
|  | | 1000 | | |  | | 549 | | | |  |  |  |  |
| Number | |  | | |  | |  | | | |  |  |  |  |
|  | |  | | |  | |  | | | |  |  |  |  |
|  | |  | | |  | |  | | | |  |  |  |  |
| Spleen - activated neutrophil | | **activated neutrophil** | | | **LDGs** | | **LDGs** | | | |  |  |  |  |
| Saline-  Ly6G+CD11b | | Pristane-  Ly6G+CD11b | | | Saline-  CD15+CD14 low | | Pristane-  CD15+CD14low | | | |  |  |  |  |
| 1828 | | 2778 | | | 877 | | 1140 | | | |  |  |  |  |
| 1627 | | 2514 | | | 759 | | 973 | | | |  |  |  |  |
| 1762 | | 2664 | | | 770 | | 980 | | | |  |  |  |  |
|  | | 1947 | | |  | | 1003 | | | |  |  |  |  |
|  | | 1173 | | |  | | 597 | | | |  |  |  |  |
|  | | 1642 | | |  | | 882 | | | |  |  |  |  |
|  | |  | | |  | |  | | | |  |  |  |  |
| NETs | |  | | |  | |  | | | |  |  |  |  |
